# A Comprehensive Approach to Predicting the Outcomes of Transsphenoidal Endoscopic Adenomectomy in Patients with Cushing’s Disease

**DOI:** 10.3390/jpm12050798

**Published:** 2022-05-16

**Authors:** Natalia V. Kuritsyna, Uliana A. Tsoy, Vladislav Y. Cherebillo, Artem A. Paltsev, Anton V. Ryzhkov, Pavel A. Ryazanov, Vladimir K. Ryzhkov, Elena N. Grineva

**Affiliations:** Almazov National Medical Research Centre, Akkuratova Str., 197341 Saint Petersburg, Russia; kuritsyna_nv@almazovcentre.ru (N.V.K.); cherebillo@mail.ru (V.Y.C.); paltsev_aa@almazovcentre.ru (A.A.P.); ryzhkov_av@almazovcentre.ru (A.V.R.); ryazanov_pa@almazovcentre.ru (P.A.R.); vlryzhkov@mail.ru (V.K.R.); grineva_en@almazovcentre.ru (E.N.G.)

**Keywords:** Cushing’s disease, hypercortisolism, transsphenoidal surgery, remission, predictors

## Abstract

Persistent and recurrent hypercortisolism after transsphenoidal endoscopic surgery (TSS) is considered to be an urgent issue prompting the search for Cushing’s disease (CD) remission predictors. The goal was to find a combination of predictors that can forecast the remission of CD after TSS. A total of 101 patients with CD who had undergone TSS were included. One year after surgery, CD remission status was evaluated. Preoperative pituitary magnetic resonance imaging (MRI) data, preoperative results of a high-dose dexamethasone suppression test (HDDST) and morning serum cortisol level collected 24 h after TSS (24 h MSeC) were compared in patients with and without remission of hypercortisolism. Remission one year after TSS was confirmed in 63 patients. CD remission predictors one year after TSS were: adenoma size ≥ 3 mm in the absence of invasive growth and the suppression of serum cortisol ≥ 74% in the HDDST, 24 h MSeC ≤ 388 nmol/L. A total of 38 patients had three favorable values of detected predictors; all of them had CD remission one year after TSS. With long-term follow-up, 36 of them remained in remission. Patients who had no one favorable predictor had no remission of hypercortisolism one year after TSS. Our data confirmed the prospects of using a combination of selected predictors to forecast CD remission after TSS.

## 1. Introduction

Cushing’s disease (CD) is a rare, severe endocrine disease caused by adrenocorticotropic hormone (ACTH)-secreting pituitary adenoma (corticotropinoma) [1,2]. Prevalence of CD is considered to be about 40 cases per million people, and incidence is 0.7 to 2.4 new cases per million per year [1,3]. The disease affects mainly young people: the average age at diagnosis is 30–40 years, and it is approximately three times more common in women [1,3,4]. Autonomous ACTH production results in chronic excess of cortisol. Severe consequences of hypercortisolism, such as cardiovascular complications, diabetes, osteoporosis and fractures, gastrointestinal ulcers, infections and thromboembolic complications lead to increased mortality, which is two times higher than in the general population [4,5,6]. Currently, the first-line treatment of CD is transsphenoidal surgery (TSS) [2,7,8]. The result of it is usually the rapid development of hypercortisolism remission. But even high-profile clinics cannot always provide the desired result after TSS in CD patients. According to a systematic review published in 2015, the median of the remission rate of CD after initial TSS was 77.9% [9]. Furthermore, the median of the recurrence rate of hypercortisolism after initially successful surgery was 11.5% [9]. It should be noted that patients with persistent or recurrent CD after TSS remain under all risks related to chronic excess of cortisol. Early identification of these patients and timely second-line treatment may reduce the negative consequences of hypercortisolism. Therefore, the detection of predictors of remission after TSS is one of the priorities in management of patients with CD. The results of various preoperative and postoperative tests are suggested as candidate remission predictors [5,7,9,10,11,12,13,14,15,16,17,18,19,20,21,22,23,24,25,26,27]. Meta-analysis of 68 studies of outcomes of pituitary surgery for CD revealed numerous predictors of remission, the strongest of which were: primary microadenomas, visible on preoperative imaging and without cavernous sinus invasion, lower preoperative 24-h urinary free cortisol and postoperative morning serum cortisol nadir <2 mg/dL (<50 nmol/L) [28]. All of these criteria reflect two aspects: the possibility of radical tumors resection (preoperative magnetic resonance imaging (MRI) data) and a successful outcome of surgery (postoperative serum cortisol tests). But there is another aspect that can affect the results of the TSS, both immediate and remote. It is a biological feature of the tumor. It is obvious that some corticotropinomas, including aggressive tumors, regardless of their size and complete resection, may relapse. We hypothesized that the results of a high-dose dexamethasone suppression test (HDDST) may reflect the biological activity of corticotropinoma. Suppression of cortisol levels during HDDST depends on the degree of pituitary adenoma autonomy, so it can characterize the risk of relapse after TSS. We also supposed that the best way to forecast the remission of CD after TSS is to use a combination of different predictors, namely, those that characterize three points: the biological potential of the pituitary tumor, the possibility of its removal during TSS and the real result of the operation.

Thus, the aim of our study was to find a combination of predictors that can forecast the remission of CD after TSS and to test its long-term effectiveness.

## 2. Materials and Methods

Patients with CD, who underwent TSS from 2010 to 2016 in the Federal Almazov National Medical Research Centre, St-Petersburg (the Almazov Centre), were included in the prospective study.

The diagnosis of CD was established according to the current guidelines [2,3]. Endogenous hypercortisolism was confirmed on the basis of high 24-h urinary free cortisol (UFC) levels, midnight serum or salivary cortisol levels and lack of cortisol suppression after low dexamethasone suppression test (LDST), >50 nmol/L. The diagnosis of ACTH-dependent syndrome was based on plasma ACTH levels > 2 pmol/L (10 pg/mL) [2,3]. For the visualization of corticotropinoma, all patients underwent dynamic pituitary magnetic resonance imaging (MRI). Bilateral cavernous and inferior petrosal sinuses sampling (BCIPSS) was performed when the MRI adenoma size was < 8 mm, using the above-mentioned technique [29,30,31,32]. HDDST was carried out before the surgery. In the cases when BCIPSS was done, the HDDST was performed after it or at least one month before the sampling. All patients with diagnosed CD underwent TSS. Surgery was done by one neurosurgery team. The diagnosis of CD was considered confirmed if the immunohistochemical (IHC) staining for ACTH was positive in the cells of the removed pituitary adenoma. In the cases when adenoma cells were not found in the removed tissue, CD was confirmed if the adrenal insufficiency and/or the need for the glucocorticoids therapy had developed. Morning serum cortisol was assessed 24 h after TSS (24 h MSeC).

All patients were examined a year after surgery and were classified into two groups: either with the remission of CD or with the persistence of CD. Remission was diagnosed if the need for glucocorticoid replacement therapy had persevered or if there was a combination of the following criteria: normalization of 24 h UFC, normalization of midnight salivary and/or midnight serum cortisol levels, and suppression of serum cortisol < 50 nmol/L after 1 mg overnight dexamethasone test [16,19]. If hypercortisolism was retained after TSS or relapsed before one-year evaluation, patients were referred to the group of CD persistence. Preoperative pituitary MRI data, the degree of cortisol suppression in the HDDST and postoperative morning serum cortisol level were compared in patients with CD remission and persistence of hypercortisolism, according to the results of a one-year examination. In patients with CD remission according to one-year post surgery examination, long-term results of TSS were evaluated at least more than 12 months after adenomectomy. The maximum follow-up period was 10 years after the operation.

For the radiological imaging of the pituitary, all patients underwent dynamic MRI of the pituitary–hypothalamic region after intravenous administration of gadolinium—111In-diethylenetriamine-pentacetic acid. Magnetom Trio A Tim 3.0 T (SIEMENS, Munich, Germany), with a detection limit of 2 mm, was used. Macroadenoma was defined as a pituitary tumor with a diameter of ≥1 cm, microadenoma—<1 cm [33]. In addition, the existence of adenoma invasive growth was estimated. For this purpose, the Knosp scale was used [34]. The growth was considered invasive if the adenoma was assigned to Knosp scale grades 2–4 [34].

HDDST was carried out according to the following protocol: serum cortisol was measured at 8:00 AM, before and after dexamethasone intake (2 mg of dexamethasone in tablets every 6 h for two days) [3].

Serum cortisol was measured with enzyme immunoassay (Roshe Diagnostic, Mannheim, Germany to the Cobas E411 analyzer, Mannheim, Germany, chemiluminescence’s technology was used). Reference range for morning cortisol was 171–536 nmol/L.

For immunnohistochemical (IHC) staining, monoclonal mouse antibodies to human ACTH were used (clone AH26, Diagnostic BioSystems, The Netherlands).

Statistical analysis was performed using the IBM SPSS Statistics 26 (New York, NY, USA). The results were presented as median (Me), interquartile range (25–75%) and minimal and maximal values (min–max). Statistical comparisons were carried out with the Mann–Whitney test for quantitative variables and the chi-square test for qualitative variables. Receiver-operating-characteristic (ROC) curve analysis was implemented to determine an optimal threshold value of serum cortisol suppression in the preoperative HDDST, preoperative MRI adenoma size and postoperative serum cortisol for prediction of CD remission after TSS. The estimate of the area under the curve (AUC) was accompanied by the 95% CI. Statistical significance was accepted for *p* < 0.05.

## 3. Results

### 3.1. Clinical Characteristics of the Patients

A total of one hundred and one patients (89 female, 12 male, median age 42 years (32; 52) (15–72), were recruited into the study. Of these, 79 patients underwent initial TSS and 22 (21.8%) repeated the procedure. In the last, the second surgery was performed a median 16.5 months (12; 22.5) (7–54) after the first one, due to the absence of remission or relapse of hypercortisolism. In 72 cases, CD was confirmed by the IHC data. In 29 patients, pituitary adenoma cells were not found in the removed tissue. A total of 19 of them developed adrenal insufficiency and the need for glucocorticoid therapy after surgery. In another ten patients, hypercortisolism persisted after TSS. In all of these patients, the TSS was repeated and CD had been confirmed after the first one (by the IHC in 7 patients and by the development of adrenal insufficiency in 3 patients). Thus, CD was confirmed in all patients.

### 3.2. Comparison of Preoperative and Postoperative Examination Data in Patients with Remission and Persistence of CD One Year after TSS

A year after TSS, CD remission was confirmed in 63 cases (62.4%). Sex, age, midnight serum cortisol, midnight salivary cortisol, 24-h urinary free cortisol, serum cortisol suppression in LDDST and morning plasma ACTH did not differ in both groups of patients; see Table 1.

#### 3.2.1. Pituitary MRI

Pituitary adenoma size was the same in patients with remission and persistence of CD; see Table 2. The incidence of CD remission after TSS did not differ in patients with micro and macroadenomas; see Table 2. The MRI sign that distinguished both groups was the presence of invasive corticotropinoma growth. Invasive growth of pituitary adenoma according to MRI data was more common in patients with CD persistence compared to patients with remission; see Table 2. For the prediction of CD remission, the sensitivity of the MRI sign «absence of invasion growth of pituitary adenoma» was 95.2%, and specificity was 55.3%. We also studied the remission rate in patients with non-invasive growth of corticotropinomas, depending on the size of the tumor.

**Figure 1:**
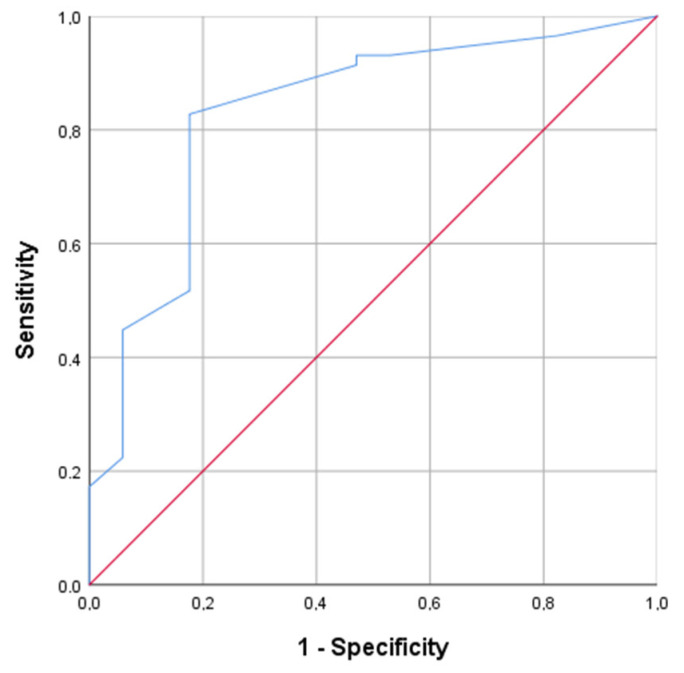
Receiver-operating-characteristic (ROC) curve of the size of a non-invasive pituitary adenoma for predicting CD remission one year after TSS. The optimal cutoff of corticotropinoma size for predicting CD remission after TSS was ≥3 mm, with the sensitivity 82.8% and the specificity 82.4% (*p* < 0.001, AUC = 0.832, 95% CI = 71.5–94.8%).

According to the results of pituitary MRI, 77 patients had non-invasive adenoma growth. Of these, 60 of them had remission and 17 had persistence of hypercortisolism. We performed the ROC analysis for detecting the optimal cutoff point of the pituitary adenoma size for predicting CD remission after TSS. According to its results, the optimal cutoff of corticotropinoma size was ≥3 mm, with the sensitivity 82.8% and the specificity 82.4% (*p* < 0.001, AUC = 0.832, 95% CI = 71.5–94.8%); see Figure 1.

#### 3.2.2. HDDST

**Figure 2:**
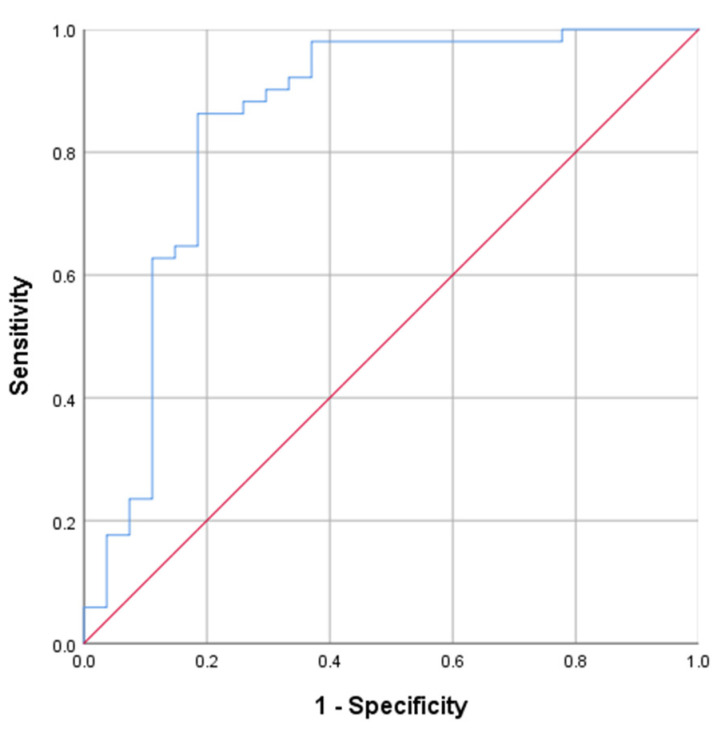
Receiver-operating-characteristic (ROC) curve of serum cortisol suppression in the HDDST for the prognosis of CD remission one year after TSS. The optimal threshold of serum cortisol suppression in the HDDST for predicting the CD remission was 74%; the sensitivity and specificity of the method were 86.3% and 81.5%, respectively (*p* < 0.001, AUC = 0.850, 95% CI = 74.4–95.6%).

HDDST was performed in 78 patients. Among them, 53 had remission and 25 had persistence of hypercortisolism one year after TSS. In 23 cases, the test was not done due to the severe course of CD (*n* = 4) and patient’s refusal. Serum cortisol suppression degree in the HDDST was higher in the remission group: 87.8% (81.8; 90.7) (21.6–96.7) vs. 55.4% (27.1; 69.7) (0.5–95.6) (*p* < 0.001). According to the ROC analysis, the optimal threshold of serum cortisol suppression in the HDDST for predicting the CD remission was 74%; see Figure 2. The sensitivity and specificity of the method were 86.3% and 81.5%, respectively (*p* < 0.001, AUC = 0.850, 95% CI = 74.4–95.6%).

#### 3.2.3. Morning Serum Cortisol Collected 24 h after TSS

**Figure 3:**
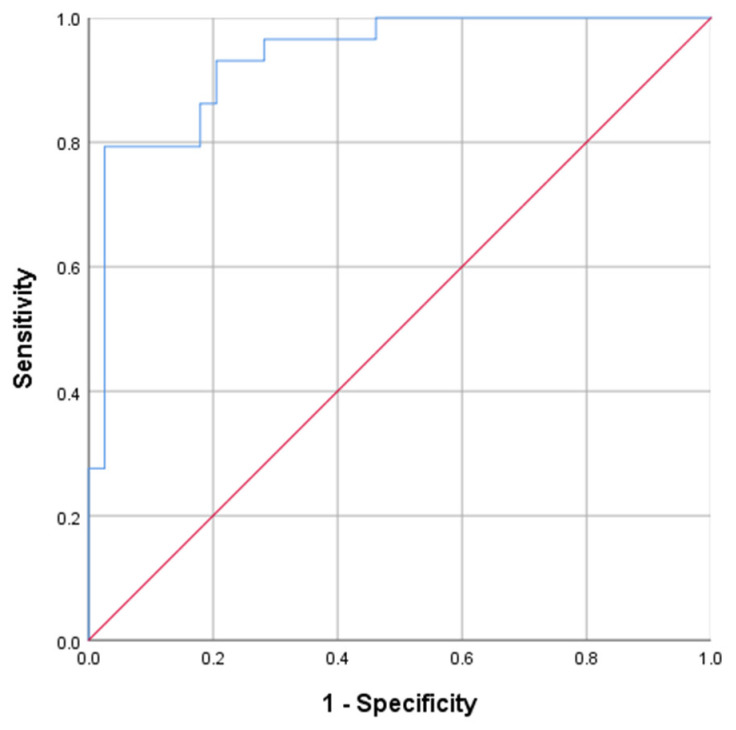
Receiver-operating-characteristic (ROC) curve of morning serum cortisol collected 24 h after TSS for prognosis of CD remission one year after TSS. The optimal threshold of 24 h MseC for predicting CD remission was ≤388 nmol/L; the sensitivity and specificity of the method were 97.4% and 79.3%, respectively (*p* < 0.001, AUC = 0.935, 95% CI = 87.7–99.2%).

The 24 h MSeC was tested in 101 patients; 63 had remission and 38 had persistence of hypercortisolism one year after TSS. The 24 h MSeC was lower in patients with CD remission: 44.1 nmol/L [23.35; 98.85] (2.5–1397) vs. 466.3 nmol/L [399.2; 619.5] (51.29–1649) (*p* < 0.001). According to the ROC analysis, the optimal threshold of 24 h MseC for predicting CD remission was ≤388 nmol/L. The sensitivity and specificity of the method were 97.4% and 79.3%, respectively (*p* < 0.001, AUC = 0.935, 95% CI = 87.7–99.2%); see Figure 3.

The specificity and sensitivity of the most common threshold values of post-operative 24 h MseC in predicting CD remission (2 mcg/dL and 5 mcg/dL (50 and 140 nmol/L) [9,10,14,15,16,25,28] were also calculated; see Table 3. All of 34 patients who had 24 h MseC ≤ 50 nmol/L preserved remission of hypercortisolism (100%). Among 67 patients with 24 h MseC > 50 mmol/L, there was CD remission in 29 patients (43.3%). Of 52 patients with 24 h MseC ≤ 140 mmol/L, CD remission developed in 48 cases (92.3%); of 49 patients with 24 h MseC > 140 nmol/L—15 cases (30.6%).

Thus, as a result of our study, three predictors of CD remission one year after TSS were determined; see Table 4. None showed 100% specificity and sensitivity in the forecast of CD remission after TSS. In order to increase the informative value of prognosis, the combination of found predictors was studied.

### 3.3. Combination of Identified Predictors for Forecast of CD Remission One Year after TSS

**Figure 4:**
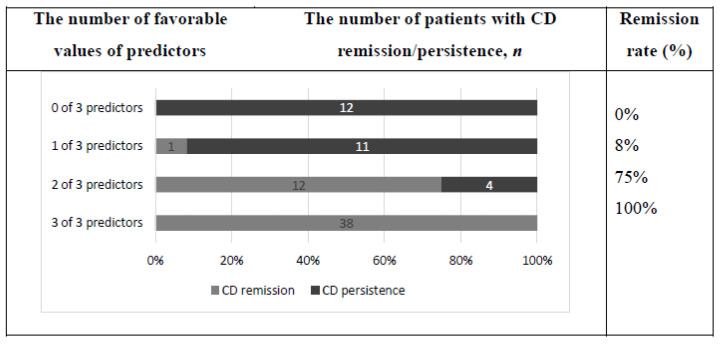
Remission rate one year after TSS depending on the number of favorable values of predictors of CD remission after surgery.

The values for all three identified predictors were obtained in 78 patients. A total of 38 out of 78 patients had favorable values of all three defined predictors of CD remission after TSS, 16 had two favorable values of predictors and 12 had one favorable value of predictors. In 12 patients, favorable values of defined predictors were absent. Remission rate, depending on the number of favorable values of defined predictors of CD remission after TSS, is presented in the Figure 4.

### 3.4. Results of the Long-Term Follow-Up

**Figure 5:**
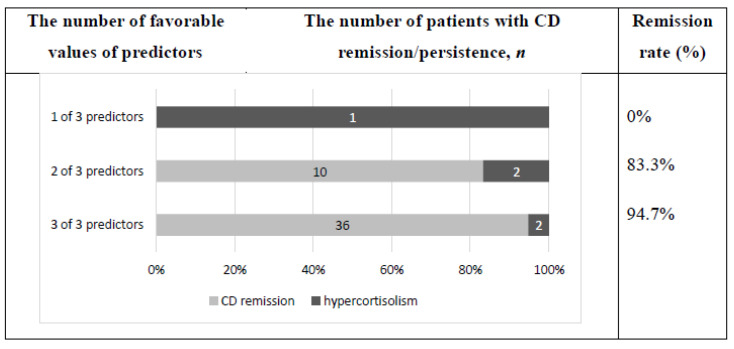
Remission rate after TSS depending on the number of favorable values of predictors of CD remission after surgery (long-term follow-up).

Patients with CD remission one year after TSS underwent a long-term follow-up. The median of the follow-up period was 41.2 months [20.5; 79.2] (15–127). Hypercortisolism relapsed in 5 (7.9%) out of 63 patients. The remission rate in the long-term follow-up group depended on the number of favorable values of CD remission predictors after TSS; see Figure 5.

## 4. Discussion

The remission rate of CD in our series of patients was 62.4% a year after TSS and 57.4% after a long-term follow-up, with a median of 41.2 months. The percentage is lower than the data of other studies, namely, the results of two meta-analyses, published by S. Petersen et al. in 2015 and by A. Stroud et al. in 2020 [9,28]. The lower remission rate might have been shown because the study group included patients after initial and after repeat TSS, and stricter remission criteria were used. One of the main conditions of the efficacy of TSS is that the neurosurgeon’s experience in our center meet the generally accepted criteria: more than 200 transsphenoidal operations in a year. We strongly believe that the outcome of TSS in patients with CD, apart from the neurosurgeon’s experience, is determined by several factors: anatomic peculiarities of the pituitary adenoma, which characterizes the possibility of its total resection, the real result of TSS, namely, the completeness of adenoma removal and the biological features of the pituitary tumor. To find the predictors of the results of TSS in CD, we analyzed the parameters that could reflect all of the above-mentioned factors.

MRI is the best tool for determining the technical feasibility of pituitary adenoma removal. The size of corticotropinoma seems to be the most obvious sign that can determine the outcome of the surgery. Indeed, in a number of studies the rate of remission was higher in patients with microadenoma than in those with macroadenoma [11,15,28]. According to meta-analysis of data from 6400 patients with CD who underwent TSS, a better remission rate was achieved in microadenomas than in macroadenomas [9]. In our study, the frequency of CD remission in patients with microadenomas and macroadenomas was the same; this corresponds to the data presented by other authors [12,26,27]. The difference can be explained by the fact that in studies showing microadenoma as a positive predictor of remission, the presence of invasive adenoma growth was not taken into consideration [11,12,15,17]. In our study, invasive growth of the adenoma, regardless of its size, was a negative predictor of CD remission after TSS. The same data were obtained in the studies published earlier [13,35]. E. Fomekong et al. presented the results of a postoperative examination of 40 patients with CD, and the remission rate in patients with macroadenomas was 92%, with microadenomas—84%. Out of the 12 patients with macroadenomas, only 1 had invasive growth of corticotropinoma, and only this particular patient did not develop CD remission after TSS [13]. Thus, we assert that invasive growth, rather than the size of the adenoma, determines the likelihood of no remission of hypercortisolism after TSS. Considering that invasive growth is mainly a feature of macroadenomas, and microadenomas rarely have invasive growth, in a large series, macroadenomas show a less favorable prognosis after TSS. According to the meta-analysis published by A. Stroud et al., visibility of adenoma on preoperative MRI is another predictor of CD remission [28]. But the visibility of adenoma strongly depends on MRI technique. That was the reason why we decided to find the minimal pituitary adenoma size favorable for CD remission after TSS. Using the ROC analysis, we obtained the optimal cutoff point for the minimal pituitary adenoma size in predicting CD remission. It is 3 mm, with sensitivity and specificity of 82.8% and 82.4%, respectively. The lower rate of CD remission in patients with pituitary adenoma less than 3 mm may be attributed to the difficulty to detect and radically remove such a small lesion. Moreover, in such cases, there is a possibility of several corticotropinomas that may not be visualized [36,37]. To our knowledge, previously the minimal pituitary adenoma size cut-off for favorable TSS result was not suggested.

In the prognosis of TSS outcome, in addition to the preoperative anatomical characteristics of pituitary adenoma, it is important to have an instrument to evaluate the nearest result of TSS. One of the markers of the completeness of corticotropinoma removal is the MSeC collected 24 h after TSS. According to the data of original studies [10,14,16,25] and meta-analysis [28], the determination of MSeC in the early postoperative period can be used to predict remission of CD. In the study of N. Hameed et al., all patients with MSeC less than 2 mcg/dL (50 nmol/L) early after TSS developed remission of hypercortisolism. Therefore, this threshold value of MSeC in the early postoperative period was indicated by the authors as the most optimal to predict CD remission after surgery [10]. According to a meta-analysis published in 2020, in 95% of patients with CD remission after initial TSS, and in 100% of patients with remission after revision TSS, the early postoperative MSeC level nadir was <2 mcg/dL (55 nmol/L) [28], indicating high specificity of this predictor. However, 46% of patients after initial TSS and 38% of patients after revision TSS also developed the remission, though they had an MSeC level nadir ≥2 mcg/dL (55 nmol/L) [28]. Thus, the sensitivity of the hypercortisolism remission predictor MSeC nadir <2 mcg/dL (55 nmol/L) is not as reliable as as its specificity. It is obvious that in clinical practice, predictor sensitivity is just as important as specificity to determine a management strategy. We therefore searched for the optimal cutoff for MSeC collected 24 h after TSS, considering not only the specificity but the sensitivity as well. We analyzed our data for the most common threshold values of MSeC in the early postoperative period. The cutoff 50 nmol/L had a specificity of 100% and a sensitivity of 53.8% in predicting of CD remission one year after surgery, and the cutoff 140 nmol/L had a specificity of 89.7% and sensitivity of 76.6%. Calculated in our study, the threshold of 24 h MSeC ≤ 388 nmol/L was higher than the most recently suggested values [9,10,14,15,16,28], but we maintain that it had an optimal ratio of specificity (79.3%) and sensitivity (97.4%). Therefore, due to this approach, we found not only the cutoff for the prediction of CD remission one year after surgery (100% specificity for the threshold 50 nmol/L) but also the cutoff for the predicting CD persistence one year after surgery (97.4% sensitivity for the threshold > 388 nmol/L).

In spite of much data that the development of adrenal insufficiency as well as early postoperative MSeC level nadir < 2 mcg/dL (55 nmol/L) are strong predictors of CD remission after TSS [9,10,14,15,16,28], some patients still develop recurrence of hypercortisolism [16,24,25]. According to the data of Yap L.B. et al., following TSS, the immediate postoperative remission (plasma levels of cortisol of <50 nmol/L or undetectable) rate was 68.5% (61/89 patients). Out of the 61 patients who were cured, 7 (11.5%) had a disease recurrence, which occurred during a mean follow-up period of 36.3 months (range: 6–60 months) [24]. This phenomenon may be explained by the biological features of the tumor. We were the first to suggest the use of HDDST to characterize it. The test normally is used to distinguish between CD and ACTH-ectopic syndrome. The basis for this test is that corticotropinomas retain the ability to express cortisol receptors and undergo ACTH and consequently cortisol suppression after high-dose dexamethasone. But the rather low sensitivity of HDDST, which is about 81–82% [3,38,39], allowed us to assume that the expression of glucocorticoid receptors or their functional activity in corticotropinomas may be different. Indeed, Mu Y-M et al. showed that the GRα mRNA levels in the adenomas in patients who showed no response to the 8-mg dexamethasone suppression test were significantly lower than those in the adenomas in patients who showed suppression [40]. Glucocorticoid receptors may not only contribute to regulation of the ACTH production but also to the control of cortocotropinoma growth via the MAPK pathway [41,42]. The more aggressive behavior of silent corticotropinomas and the development of pituitary macroadenoma in Nelson syndrome [43] may confirm the hypothesis of the involvement of glucocorticoids in the negative regulation of corticotropinoma growth. All of these data allowed us to suggest that the degree of cortisol suppression in HDDST reflects not only the autonomy of ACTH hyperproduction but also the potential for corticotropinoma recurrence or continued growth and may be the prognostic marker of CD remission after TSS. According to our data, the degree of suppression of serum cortisol in HDDST was higher in patients with CD remission one year after surgery compared with those who retained hypercortisolism. The optimal threshold of serum cortisol suppression in the HDDST for predicting the CD remission was 74%.

None of the identified criteria had perfect specificity and sensitivity to the prediction of CD remission. We hypothesized that the use of a combination of predictors may optimize the prognosis. In fact, within the one-year follow-up period, 100% of patients who had three favorable predictors remained in remission of CD. In addition, all patients who had no one favorable predictor had persistence of hypercortisolism. The results of long-term follow-up confirmed the efficacy of such a comprehensive approach. Only two patients among those who had three favorable predictors developed relapse of hypercortisolism: 4 and 7 years after TSS. This highlights the fact that it isn’t just glucocorticoid receptor status that determines the biological potential of the tumor. Furthermore, HDDST is not effective as a predictor of long-term hypercortisolism remission if CD develops due to hypothalamic dysfunction. In this case, the cause of the hypercortisolism recurrence may be de novo-developed corticotropinoma with new biological potential.

The limitations of our research included a small sample of patients. That can be explained by the timing of the study and the possibility of long-term follow-up. Not all patients underwent HDDST. The test is not currently mandatory in the diagnostic protocol for ACTH-dependent hypercortisolism in the Almazov Center. Some patients refused to undergo the test; in some patients, it was not done due to the severity of the patient’s condition. The need to use the HDDST only as a predictor of the TSS result is controversial. But the relevance of assessing the biological potential of a tumor in predicting the outcome of TSS is obvious.

## 5. Conclusions

Our data confirmed the prospects of a comprehensive approach to predicting remission of CD after TSS. In day-to-day management of CD patients, not only specificity but also the sensitivity of the predictor is important, and that must be taken into account when choosing threshold values. All patients with CD need a long-term follow-up. Studies aimed at finding a new way to assess corticotropinoma’s biological potential and applications in real clinical practice are needed.

## Figures and Tables

**Table 1:** Preoperative characteristic and comparison of patients with remission and persistence of CD one year after TSS.

	Remission (N = 63)	Persistence (N = 38)	*p*
Male/female, *n*	5/58	7/31	0.208 (χ^2^ = 1.588)
Age, years Me (25%; 75%) (min–max)	43 (33; 53) (21–68)	38 (28.75; 47.5) (15–72)	0.336
Midnight serum cortisol, nmol/L Me (25%; 75%) (min−max)	496.6 (381.7; 750.2) (274.5−1453)	605 (436; 754) (316−1006)	0.156
24-h UFC, nmol/24-h Me (25%; 75%) (min–max)	588.96 (419.1; 921.1) (66–6406)	762.4 (432; 2096.5) (156.6–8740)	0.325
Morning plasma ACTH, pg/mL Me (25%; 75%) (min–max)	75.7 (46.2; 91.4) (12.5–241.9)	56.99 (47.1; 78) (19.64–213)	0.201
Midnight salivary cortisol, nmol/L Me (25%; 75%) (min–max)	14.5 (8.6; 22.3) (5.42–42.11)	11.76 (8.75; 16.5) (3.8–44.3)	0.443
Serum cortisol (LDDST), nmol/L Me (25%; 75%) (min–max)	306.9 (163.5; 537.2) (13.24–883)	477 (368.8; 584.2) (63.97–770.3)	0.085

24-h UFC—24-h urinary free cortisol; ACTH—adrenocorticotropic hormone; LDDST—Low-dose dexamethasone suppression test.

**Table 2:** Preoperative MRI data in patients with remission and persistence of CD one year after TSS.

	Remission (N = 63)	Persistence (N = 38)	*p*
Pituitary adenoma size, mm Me (25%; 75%) (min–max)	6 (4;8) (2.2–22)	5.5 (3; 8.5) (2–32)	0.31
Age, years Me (25%; 75%) (min–max)	43 (33; 53) (21–68)	38 (28.75; 47.5) (15–72)	0.336
Microadenoma/macroadenoma, *n*	52/11	30/8	0.655 (χ^2^ = 0.2)
MRI-invisible adenoma, N	2	3	0.584 (χ^2^ = 0.3)
Invasive growth, *n*	3	21	<0.001 (χ^2^ = 33.4)

Me—median, MRI—magnetic resonance imaging.

**Table 3:** Sensitivity and specificity of morning serum cortisol collected 24 h after TSS assessment, depending on its threshold levels in predicting CD remission after TSS.

24 h MSeC	Sensitivity (%)	Specificity (%)
50 nmol/L	53.9%	100%
140 nmol/L	76.2%	89.5%
388 nmol/L	97.4%	79.3%

24 h MSeC—morning serum cortisol collected 24 h after transsphenoidal surgery, TSS—transsphenoidal surgery.

**Table 4:** Sensitivity and specificity of defined CD remission predictors.

Predictor	Sensitivity (%)	Specificity (%)
Preoperative MRI data (none-invasive adenoma size ≥ 3 mm)	82.8%	82.4%
Preoperative HDDST (serum cortisol suppression ≥ 74%)	86.3%	81.5%
24 h MSeC ≤ 388 nmol/L	97.4%	79.3%

MRI—magnetic resonance imaging, HDDST—high-dose dexamethasone suppression test, 24 h MSeC—morning serum cortisol collected 24 h after transsphenoidal surgery.

## Data Availability

Data supporting reported results can be requested from corresponding author.
